# Retinal dehydrogenase 5 (RHD5) attenuates metastasis via regulating HIPPO/YAP signaling pathway in Hepatocellular Carcinoma

**DOI:** 10.7150/ijms.46091

**Published:** 2020-07-19

**Authors:** Hao Hu, Liang Xu, Shao-Ju Luo, Ting Xiang, Yan Chen, Zhi-rui Cao, Yu-jian Zhang, Zhuomao Mo, Yongdan Wang, Dong-fang Meng, Ling Yu, Li-zhu Lin, Shi-Jun Zhang

**Affiliations:** 1 Department of Oncology, First Affiliated Hospital of Guangzhou University of Chinese Medicine, Guangzhou (510407), China.; 2 The First Affiliated Hospital, Sun Yat-Sen University, Guangzhou 510080, Guangdong, P. R. China.; 3 The Sixth Affiliated Hospital, Sun Yat-Sen University, Guangzhou 510080, Guangdong, P. R. China.; 4 Department of Chinese Medicine, the Third Affiliated Hospital, Sun Yat-Sen University, Guangzhou 510630, Guangdong, P. R. China.; 5 Department of Radiation Oncology, Shandong Cancer Hospital and Institute, Shandong First Medical University and Shandong Academy of Medical Sciences, Jinan, 250117, P. R. China.

**Keywords:** Retinal dehydrogenase 5, Hepatocellular carcinoma, Metastasis, Yes-associated protein

## Abstract

Retinal dehydrogenase 5 (RDH5) is an important enzyme in the visual cycle. Several studies have reported that the RDH family may play crucial roles in tumor prognosis. However, the role of RDH5 in tumor prognosis is still unclear. We examined the mRNA level of RDH5 by using q-PCR in hepatocellular carcinoma (HCC) and adjacent non-cancerous tissues. The proliferation rate of HCC cells was detected by MTS assay, and the invasive ability was examined by transwell and scratch wound assays. The YAP protein localization and expression were visualized by immunofluorescence in two different cell lines. CpG islands in the promoter region were predicted by using the methprimer database. Clinical characteristics of a patient cohort data came from The Cancer Genome Atlas database. RDH5 was significantly downregulated in hepatocellular carcinoma tissues, and low RDH5 expression was associated with metastasis and poor patient prognosis. Functional assays revealed that the RDH5 promoter is methylated in HCC cell lines. Moreover, overexpressing RDH5 can suppress metastasis by reversing the epithelial-mesenchymal transition (EMT) process, and RDH5 also inhibits cell proliferation in HCC cell lines. Furthermore, suppressing RDH5 can activate the Hippo/YAP signaling pathway and promote the nuclear translocation of YAP. Clinical data demonstrated that RDH5 is an independent prognostic factor in HCC. In our study, we provided the first evidence that RDH5 plays a crucial role in suppressing proliferation and metastasis, and the RDH5 promoter is methylated in hepatocellular carcinoma. And as an important regulator, RDH5 can suppress the Hippo/YAP signaling pathway. Taken together, it revealed that RDH5 might be a potential therapeutic target in HCC patients.

## Introduction

Hepatocellular carcinoma (HCC) is the sixth most common primary malignancy and the third leading cause of cancer-related mortality in the world 1, 2. In East Asia, especially China, the hepatitis B virus (HBV) infection is the main etiological factor for HCC 3. Nowadays, metastasis is the most common reason for poor prognosis in HCC patients 4, 5. Although surgical resection 6, liver transplantation 7, 8, and other clinical methods are used to treat HCC, a large number of patients with long distant metastasis have not opportunity for radical resection. Therefore, there is an urgent need to find valuable biomarkers and potential therapeutic targets in HCC patients.

Currently, serum alpha-fetoprotein (AFP) 9, 10, imaging technologies 11, and histology 12 are three predominant methods for HCC screening. However, clinically, the limitation of AFP testing is lacking high sensitivity and specificity, especially in patients without typical AFP changes. Imaging technologies can only effectively diagnose the disease when the tumor is huge, and it failed to detect tumors that smaller than 1 cm in diameter. Histological diagnosis depends on the availability of tissue biopsy samples, and this method carries the risk of implantation metastasis.

Retinal dehydrogenase 5 (*RDH5*) belongs to the RDH enzyme family, and it is an important enzyme in the visual cycle that catalyzes the oxidation of 11-cis-retinol to 11-cis-retinal 13, 14. Human *RDH5* is encoded by the* RDH5* gene, which maps to chromosome 12q13-q14 and encodes a 32 kDa protein with 318 amino acids 15, 16. According to the literature, the expression dysregulation, nonsense mutations, and DNA methylation of RDH5 could easily result in night blindness. Some reports have demonstrated *RDH5* was dysregulated in many different cancers. In colorectal carcinoma, *RDH5* was significantly decreased in the mRNA level, leading to diminished all-trans-retinoic acid biosynthesis and thus contributing to the progression of colorectal cancer by affecting cell growth and differentiation 17. Exome sequencing of early gastric carcinoma (EGC) patient samples revealed that* RDH5* mutations might be involved in early carcinogenesis 18. Another study reported *RDH5* were hypomethylated and overexpressed in papillary thyroid carcinoma compared to matched non-neoplastic adjacent tissues 19. However, whether *RDH5* expression is involved in HCC recurrence and metastasis remains unclear.

The Hippo pathway is an evolutionarily conserved signaling module that plays a critical role in liver size control and tumorigenesis 20, 21. In humans, the Hippo pathway is a kinase cascade wherein macrophage-stimulating protein 1/2 (MST1/2) and large tumor suppressor kinase 1/2 (LATS1/2), it could be phosphorylated and activated, and result in the phosphorylation and inactivation of Yes-associated protein (YAP) 22. When YAP is retained in the cytoplasm, it is degraded rapidly; when it enters the nucleus, it transcriptionally activates anti-apoptosis genes 23. Nevertheless, the link between *RDH5* and the Hippo pathway has not been reported.

In this study, we aimed to document the clinical significance of *RDH5* and identify potential biomarkers for metastatic HCC patients and cell lines. Every effort was made to identify a novel therapeutic target for HCC patients.

## Materials and methods

### Cell lines and cell culture

The human hepatocellular carcinoma cell lines Hep 3B and SK-Hep-1 were obtained from the Institute of Biochemistry and Cell Biology (Shanghai, China) and were maintained in Dulbecco's modified Eagle's medium supplemented with 10% FBS at 37°C and 5% CO2. The cancer cell lines were authenticated using short tandem repeat profiling, and the cells were not cultured for more than two months.

### Patients and specimens

Fifteen matched human HCC tissues and adjacent non-cancerous tissues were collected and analyzed by quantitative real-time PCR. These samples were obtained by surgical resection from patients with HCC in May and July of 2016 and stored immediately in liquid nitrogen for subsequent analysis 24. All of our studies were approved by the committee of the Ethical Review of Research at Sun Yat-Sen University Cancer Center.

### Experimental Animals

Totally 10 specific pathogen-free (SPF) C57BL/6 mice (male; weight, 23±2 g) were purchased from the Animal Experimental Center of the Guangdong province. The Diethylnitrosamine (N-nitrosodiethylamine, DEN, CAS: 55-18-5) was purchased from Sigma, and the reserpine injection was purchased from the Aladdin (R101672). All of the C57BL/6 mice accepted 25 mg/kg DEN with intraperitoneal injection in the 14 days, and another DEN injection performed in the 30 days for inducing HCC have been developed, it will take 10 months to develop HCC. The “3R” principles with humanism care to the experimental animals was performed strictly and all of our studies obtained approval from the research ethics committee at Sun Yat-Sen University.

### Plasmid and small interfering RNA (siRNA) transfections

*RDH5* was cloned into the pcDNA3.1 vector. All transfections were performed using Lipofectamine 2000 according to the manufacturer's instructions. The empty pcDNA3.1 vector was used as the control, pcDNA3.1-RDH5 and vector control were transfected into the SK-Hep-1 cell. The targets of the* RDH5* siRNAs were 5'-AAAUGAGCUACAUCCCGCCUCAG-3' (si#1) and 5'-ACCCUGUUGGAUAUCACUGAUTT-3' (si#2). The negative control (NC) siRNA was purchased from KEYGENTEC. All transfections were performed using Lipofectamine RNAiMAX (Invitrogen) according to the manufacturer's instructions. The gene-silencing or plasmid transfection efficacy was evaluated 48 h after transfection by qPCR and immunoblotting.

### Immunoblotting

Cells were lysed in RIPA buffer containing a protease (TargetMol, CC0001) and phosphatase (TargetMol, CC0004). Protein concentrations were assessed using the BCA Protein Assay Kit (Pierce Biotechnology). Equal amounts of protein mixed with sample loading buffer (5X; Beyotime) were separated by SDS-PAGE and transferred to polyvinylidene difluoride membranes (Millipore). After blocking with 5% skim milk or BSA in Tris-buffered saline Tween-20 (TBST), the membranes were incubated with primary antibodies overnight at 4°C and then with horseradish peroxidase-conjugated secondary antibodies at room temperature for 1 h. The protein bands were visualized using a chemiluminescence kit (Pierce). GAPDH was used as a control for equal protein loading. The primary antibodies included anti-RDH5 (Abcam, ab200197), anti-GAPDH (Proteintech, 60004-1-1g), anti-YAP1 (Proteintech, 13584-1-AP), and anti P-YAP antibodies (CST, 13008), anti-mouse and anti-rabbit peroxidase-conjugated secondary antibodies were used 25, 26. All experiments were independently repeated at least three times.

### Real-time quantitative PCR analysis

The expression level of each target gene was determined by RT-PCR. Total mRNA was isolated from hepatocellular carcinoma tissues using Trizol reagent (Invitrogen, CA, USA). The internal control for measuring the *RDH5* expression level was GAPDH. The relative expression levels of the target genes were estimated by calculating two to the power of ΔCt (Ct of GAPDH minus Ct of the target gene), and the experiments were repeated three times 27, 28. The following PCR primer sequences were used in our research:

- RDH5, forward, 5'-CTGTGACCAACCTGGAGAGTCT-3',
- RDH5, reverse, 5'-GATGCGCTGTTGCATTTTCAGGT-3',
- GAPDH, forward, 5'-GTCTCCTCTGACTTCAACAGCG-3',
- GAPDH, reverse, 5'-ACCACCCTGTTGCTGTAGCCAA-3'.

### Immunofluorescence

For immunofluorescence analysis of cultured cells, 3×10^4^ HCC cells were seeded on coverslips, fixed with 4% paraformaldehyde (PFA) for 20 min at room temperature, blocked with 5% BSA in PBS + 0.1% Triton X-100 (PBST), incubated with the primary antibody at 4°C overnight, washed with PBST, and incubated with secondary antibody at room temperature. Mounting medium containing 4',6-diamidino-2-phenylindole (DAPI) was placed on the slides, and the staining was imaged using an OLYMPUS FV1000 microscope 29.

### DNA methylation

Hep 3B and SK-Hep-1 cells were treated for 48 h with 0, 2, 5, and 10 mM 5-Aza. After 2 or 3 days, cells were harvested, and total RNA was extracted from the cultured cell lines. GAPDH was used as the loading control.

### Cell growth curve

MTS assay was performed to determine cancer cell growth and viability. Briefly, 1000 cells/200 ml of medium were seeded into a 96-well plate (Corning) and cultured without FBS to maintain synchronization and avoid too rapid growth in one week. At various time points after seeding, the cells in each well were stained with 20 ml of MTS (Promega, G3580) for 3 h, and the OD490 was determined with a microplate reader. All experiments were independently repeated at least three times.

### Scratch wound assay

Cells were digested after transfection 48 hrs and seeded in a 6-well plate. A scratch wound assay was performed by generating a wound in the center of each well in a 6-well plate with a sterile 200 µl pipette tip. The unattached cells were removed by washing with PBS, and serum-free medium or medium with 3% FBS was added. Subsequently, cells were observed with an inverted microscope at 0, 20, or 24 h.

### The Cancer Genome Atlas (TCGA) data analysis

Publicly available human HCC datasets in the TCGA were analyzed. The mRNA expression profiles downloaded from the TCGA portal (https://cancergenome.nih.gov/) included 49 non-neoplastic adjacent tissue samples and 374 tissue samples. Excluding those with unavailable clinicopathological features, 307 cases were included in the cohort to analyze the clinical characteristics 24.

### Statistical analysis

All data in this study were evaluated with SPSS 21.0 software (SPSS Inc., Chicago, USA) and GraphPad Prism (GraphPad software). All data are shown as the mean ± standard deviation. The results were evaluated using the Student's t-test. Survival analysis was performed using the Kaplan-Meier method and log-rank test. The relevance of the relationship between *RDH5* expression and clinicopathological parameters was determined using the chi-square test. Cox proportional hazards regression models were constructed to evaluate the data, and *p*<0.05 indicated a statistically significant difference.

## Results

### RDH5 is downregulated in HCC tissues

**Figure 1:**
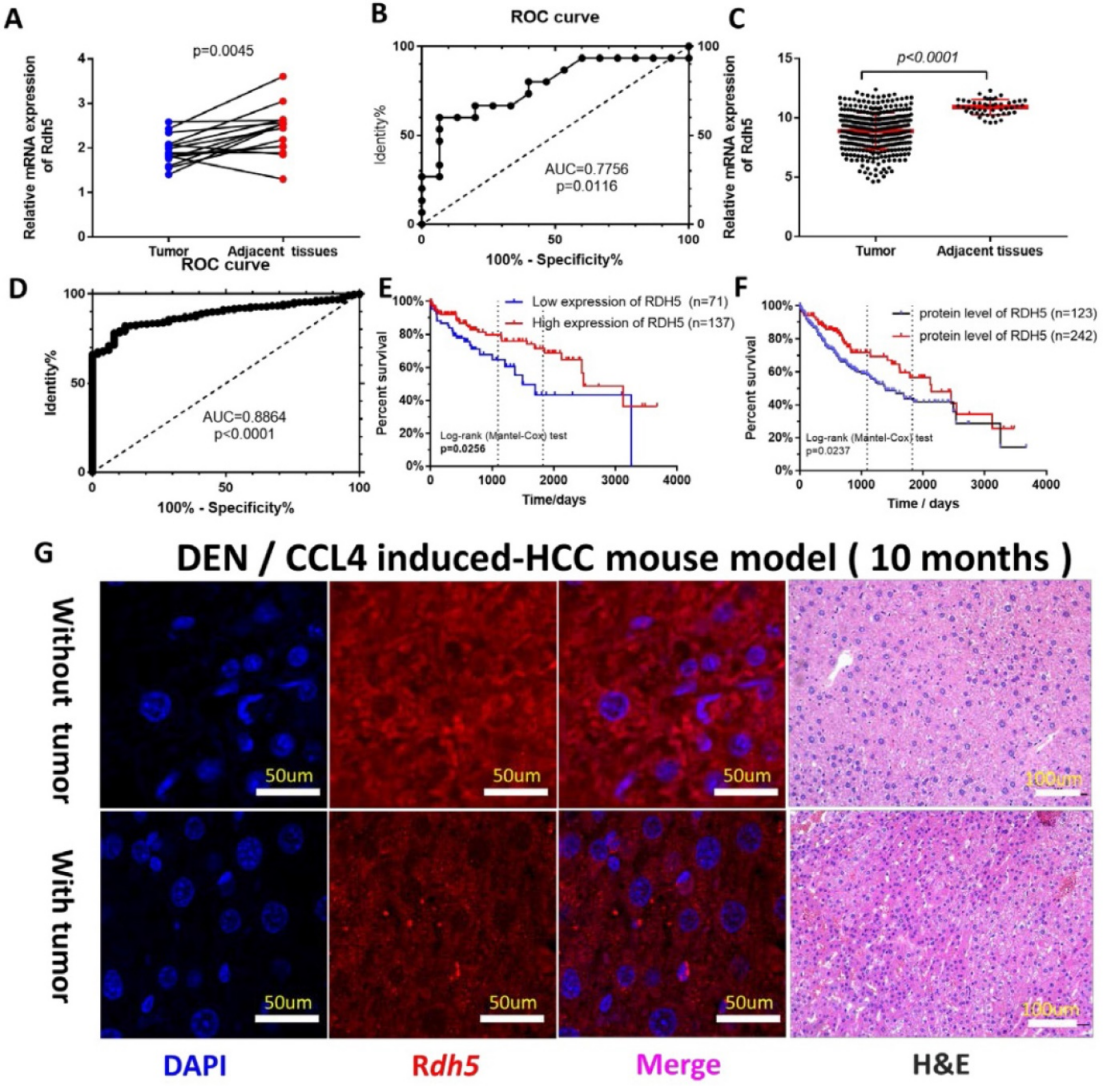
** RDH5 is down-regulation in HCC tissues and its expression is associated with overall survival**. In our cohort **(A)** and TCGA data** (B)**, mRNA expression of RDH5 is significantly higher in adjacent tissues than HCC tissues. The area under the curve of the ROC curve is 0.7756 in our tissue samples **(C)** and 0.8864 in the TCGA database** (D)** respectively, p<0.05. Overall survival in mRNA expression **(E)**. or the protein level **(F)**, by Kaplan-Meier analysis. **(G)** RDH5 is down-regulation in mouse HCC tissues by Immunofluorescence technique.

To investigate the possible role of *RDH5* in HCC progression, a real-time quantitative PCR assay was used to detect gene expression in 15 paired tumor tissues and adjacent non-cancerous tissues. The results showed *RDH5* was highly expressed in adjacent non-cancerous tissues than in HCC tissues **(**p=0.0045) (Fig. 1A). The Receiver operating characteristic (ROC) curves were used to estimate the value of *RDH5* in mRNA expression level, which yielded an area under the curve (AUC) of 0.7756 (Fig. 1C). Similar results from the TCGA database was shown that the expression of *RDH5* was significantly downregulated in tumor tissues (p<0.001) (Fig. 1B), with an area under the curve of 0.8864 (n=422) (Fig. 1D). Protein expression was visualized by immunofluorescence, which shown that downregulating RDH5 in DEN and CCL4 induced-mouse hepatocellular carcinoma (Fig. 1G). Therefore, we hypothesized that RDH5 plays a critical role in HCC tumorigenesis and progression.

### RDH5 is associated with the metastasis and prognosis in HCC patients

As the increased RDH5 mRNA level in adjacent non-cancerous tissues were clearly shown, it was necessary to elucidate the correlation between RDH5 expression and clinical survival. To further determine whether the downregulation of RDH5 is associated with poor prognosis in HCC patients, 307 HCC specimens were analyzed for clinicopathological features: 63% (193 of 306) of HCC patients had low RDH5 expression, and the remaining 37% (113 of 306) had high RDH5 expression. The clinical characteristics of these patients are shown in Table 1. Clinicopathological parameters included gender, age, Child-Pugh classification, AFP, fibrosis, grade, TNM stage, clinical-stage, vascular stage, and year of diagnosis. Only the M stage was relevant to RDH5 expression, and lower expression of RDH5 was associated with much more metastasis in HCC patients.

To further explore the relationship between *RDH5* expression and survival, we conducted an overall survival analysis by the Kaplan-Meier method. We found out in the low RDH5 level group, the estimated 3- and 5-year OS rates were 64.62% and 43.54%, respectively, for the high *RDH5* level group was 79.70% and 71.46%, respectively (Fig. 1E). Disease-free survival was not affected by *RDH5* expression (data not shown). In the cohort collected from the human protein alter (THPA), the results showed that patients with high *RDH5* expression had a longer mean survival time than patients with low *RDH5* expression, and lower level of the protein RDH5 may be contributed to the poor prognosis in overall survival (Fig. 1F). Taken together, these analyses revealed that a high RDH5 level in HCC significantly correlated with poor patient outcomes.

### RDH5 suppresses cell migration by reversing epithelial-mesenchymal transition in hepatocellular carcinoma cell lines

**Figure 2:**
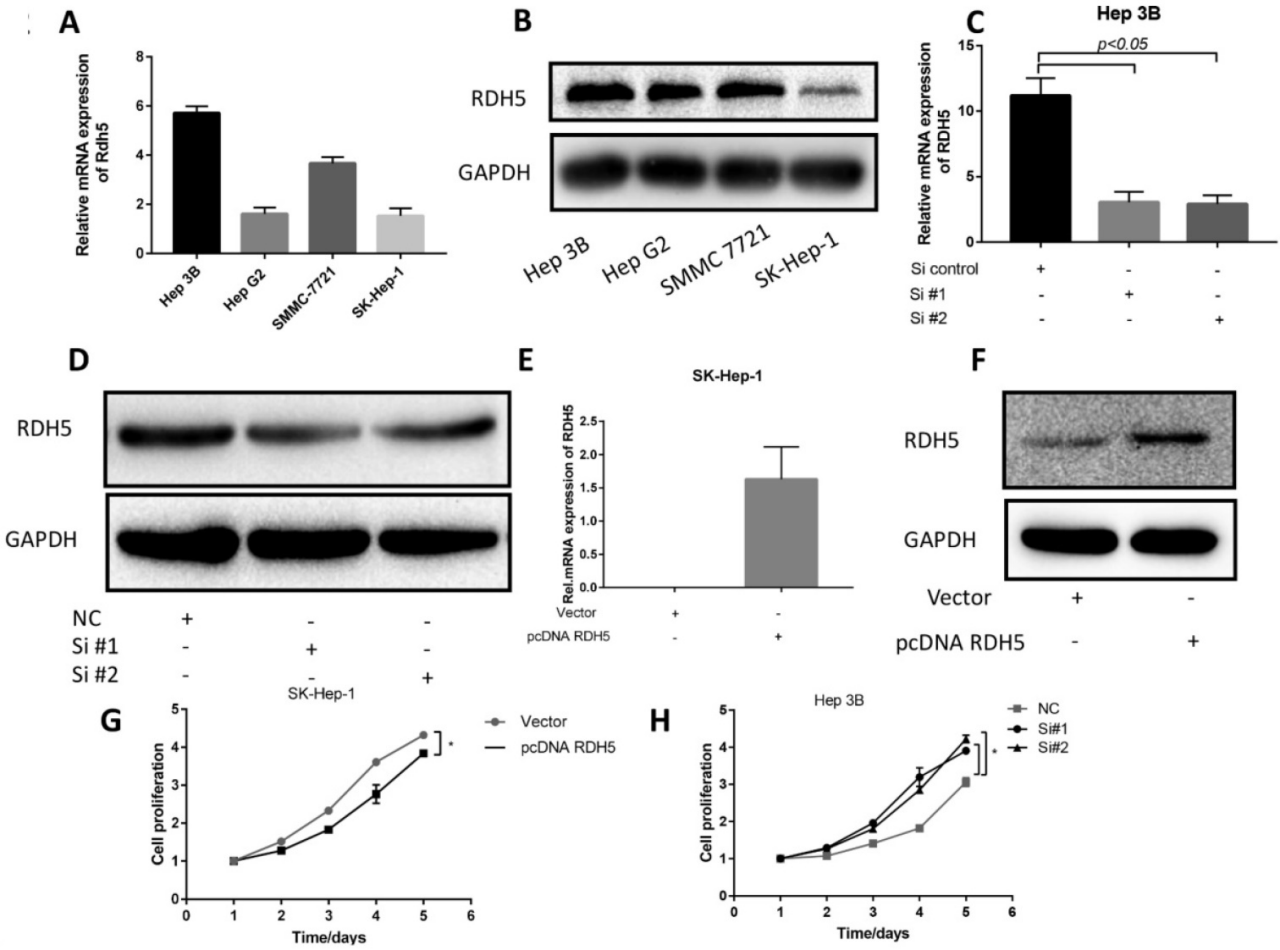
** RDH5 expression in the HCC cell lines and promoted proliferation in HCC cell lines. (A)** The mRNA level of RDH5 (normalized to GAPDH) in 4 HCC cell lines was confirmed by qPCR, showing that RDH5 was significantly lower in Hep G2 and SK-Hep-1 than Hep 3B and SMMC-7721. **(B)** The protein level of RDH5 in 4 HCC cell lines was confirmed by immunoblotting. Overexpression of RDH5 in SK-Hep-1 was determined by qPCR**(C)** and immunoblotting **(D)**, and GAPDH was used as the loading control. The transient suppression of RDH5 in Hep 3B was determined by qPCR **(E)** and by immunoblotting **(F)**.GAPDH was used as the loading control. Up-regulation of RDH5 expression in SK-Hep-1 suppressed cell proliferation ability by MTS assay **(G),** cell proliferation ability was promoted after RHD5 was silenced in Hep 3B **(H)**.

**Figure 3:**
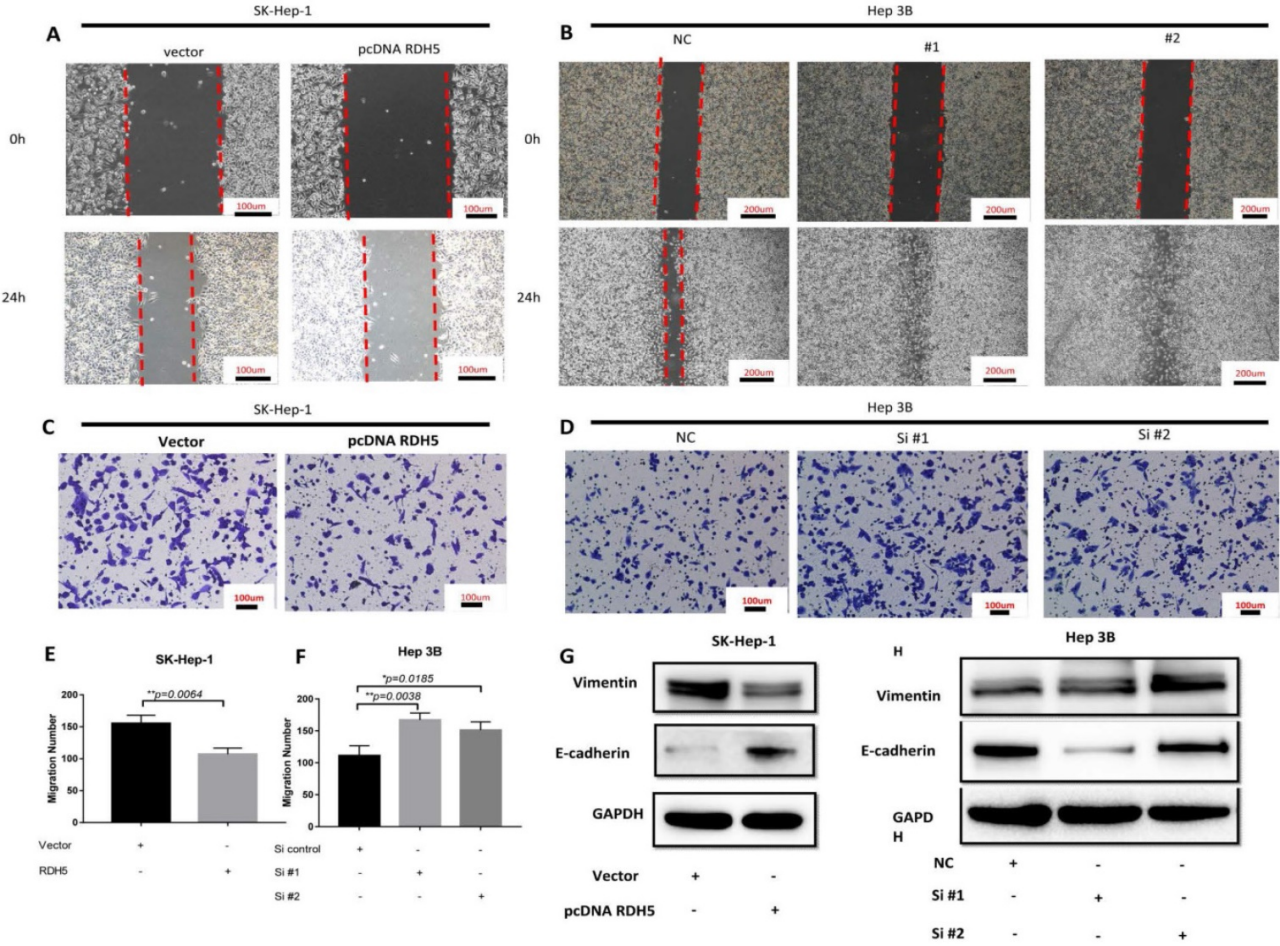
** RDH5 promoted cell migration by reversing the epithelial-mesenchymal transition.** A Wound-scratch assay was performed to determine the effect of RDH5 on cell migration. **(A)** Overexpression of RDH5 in SK-Hep-1 inhibited the migration ability, and **(B)** transient suppression of RDH5 in Hep 3B dramatically promoted the wound healing ability. Up-regulation of RDH5 expression in SK-Hep-1 suppressed the migration ability by transwell assay **(C, E),** suppression of RDH5 in Hep 3B promoted migration ability by transwell assay** (D, F)** The levels of mesenchymal marker such as vimentin was decreased and level of epithelial protein E-cadherin was elevated in the RDH5-overexpressing SK-Hep-1 cell **(G),** the levels of epithelial marker E-cadherin was decreased and level of mesenchymal protein vimentin was elevated in the RDH5-silencing Hep 3B **(H).**

Upon investigating the mRNA and protein levels of RDH5 in HCC cell lines, decreased RDH5 expression was observed in Hep 3B and SMMC-7721 cells compared with SK-Hep-1 and HepG2 cells (Fig. 2A, B). To explore the relationship between RDH5 and metastasis *in vitro*, we transiently overexpress RDH5 in SK-Hep-1 cells and silence RDH5 in Hep 3B cells (Fig. 2C-F). We generated cell growth curves, which revealed that HCC cell proliferation was suppressed by overexpressing RDH5 and increased by silencing RDH5 (Fig. 2G, H). We applied to wound healing and transwell assays to investigate the migratory ability of Hep 3B and SK-Hep-1 cells and found that overexpressing RDH5 dramatically inhibited wound healing ability (Fig. 3A, B) and reduced the cell migration ability (Fig. 3C-H). Furthermore, we performed the western blotting analysis to investigate epithelial-mesenchymal transition (EMT) relevant markers. Overexpressing RDH5 resulted in decreased levels of mesenchymal markers such as vimentin and elevated levels of epithelial proteins such as E-cadherin (Fig. 3G). Silencing RDH5 resulted in increased levels of mesenchymal marker vimentin and decreased levels of the epithelial protein E-cadherin in Hep 3B cells (Fig. 3H). These results proved that RDH5 can inhibit cell migration by reversing epithelial-mesenchymal transition *in vitro*.

### RDH5 expression is regulated by DNA methylation in Hepatocellular carcinoma cells

**Figure 4:**
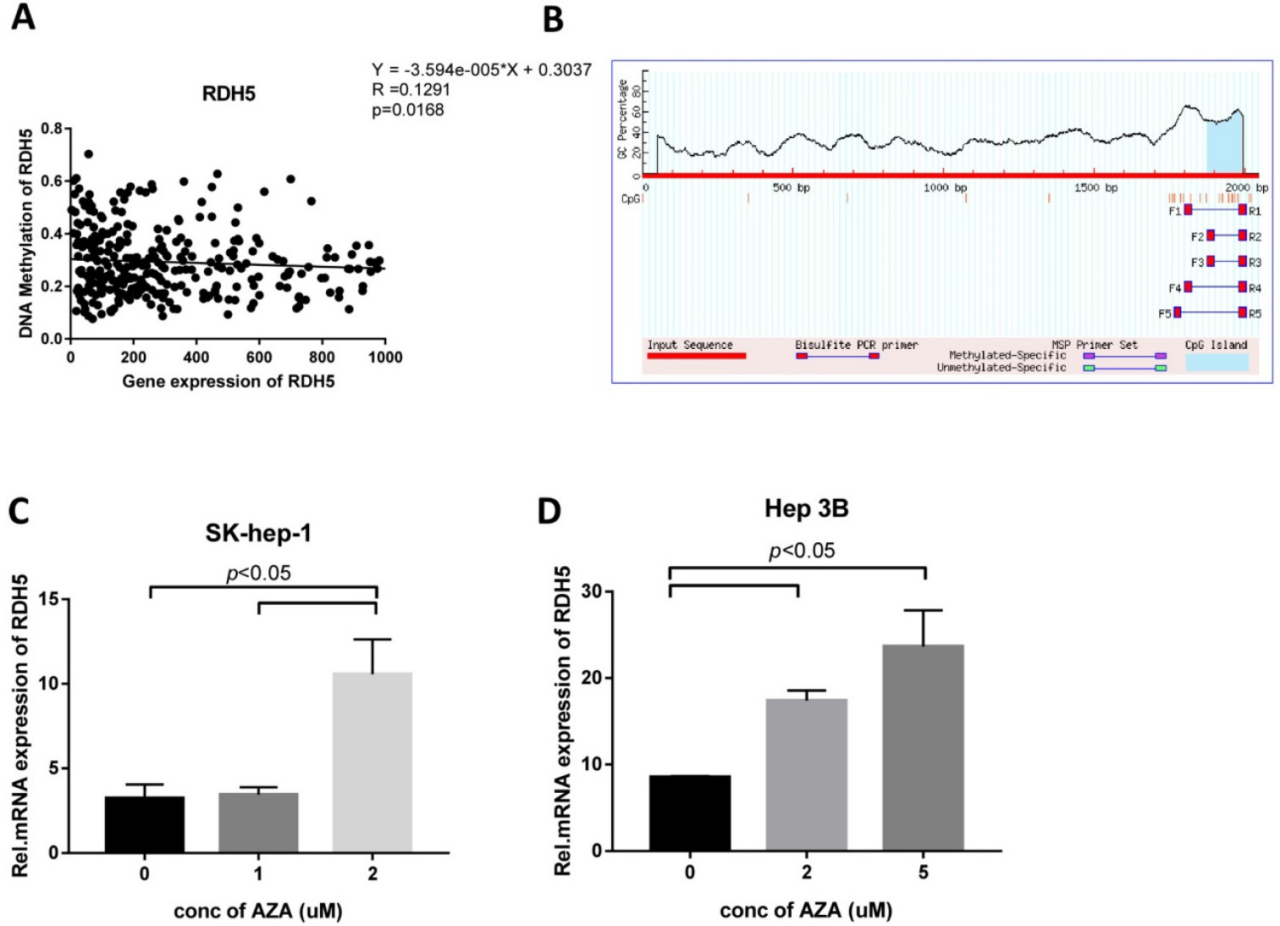
** RDH5 expression is regulated by DNA methylation (A)** RDH5 mRNA expression was positively correlated with RDH5 DNA methylation in the TCGA database, and **(B)** CpG islands in the promoter region was predicted in the biomedical website. SK-Hep-1**(C)** and Hep 3B **(D)** cells were subjected to a 72-h treatment with 0,1,2,5 doses of 5-Aza. After RNA extraction, RDH5 mRNA was quantified by qPCR.

To explore the role of DNA methylation in RDH5 expression, CpG islands in the promoter region were predicted by using http://www.urogene.org/methprimer/, and RDH5 was shown to be silenced by DNA methylation in the TCGA analysis (Fig. 4A, B). Interestingly, the inhibition of DNA methyltransferase by 5-aza-2-deoxycytidine (DAC) at concentrations of 0, 1, 2 and 5 µM suppressed DNA methylation levels and increased the expression of RDH5 in Hep 3B and SK-Hep-1 cells (Fig. 4C, D). The increase of RDH5 expression after treatment with DAC was consistent with the baseline DNA methylation levels in the Hepatocellular carcinoma cell line.

### RDH5 suppresses the EMT process via inhibiting Hippo/YAP signaling pathway in hepatocellular carcinoma

**Figure 5:**
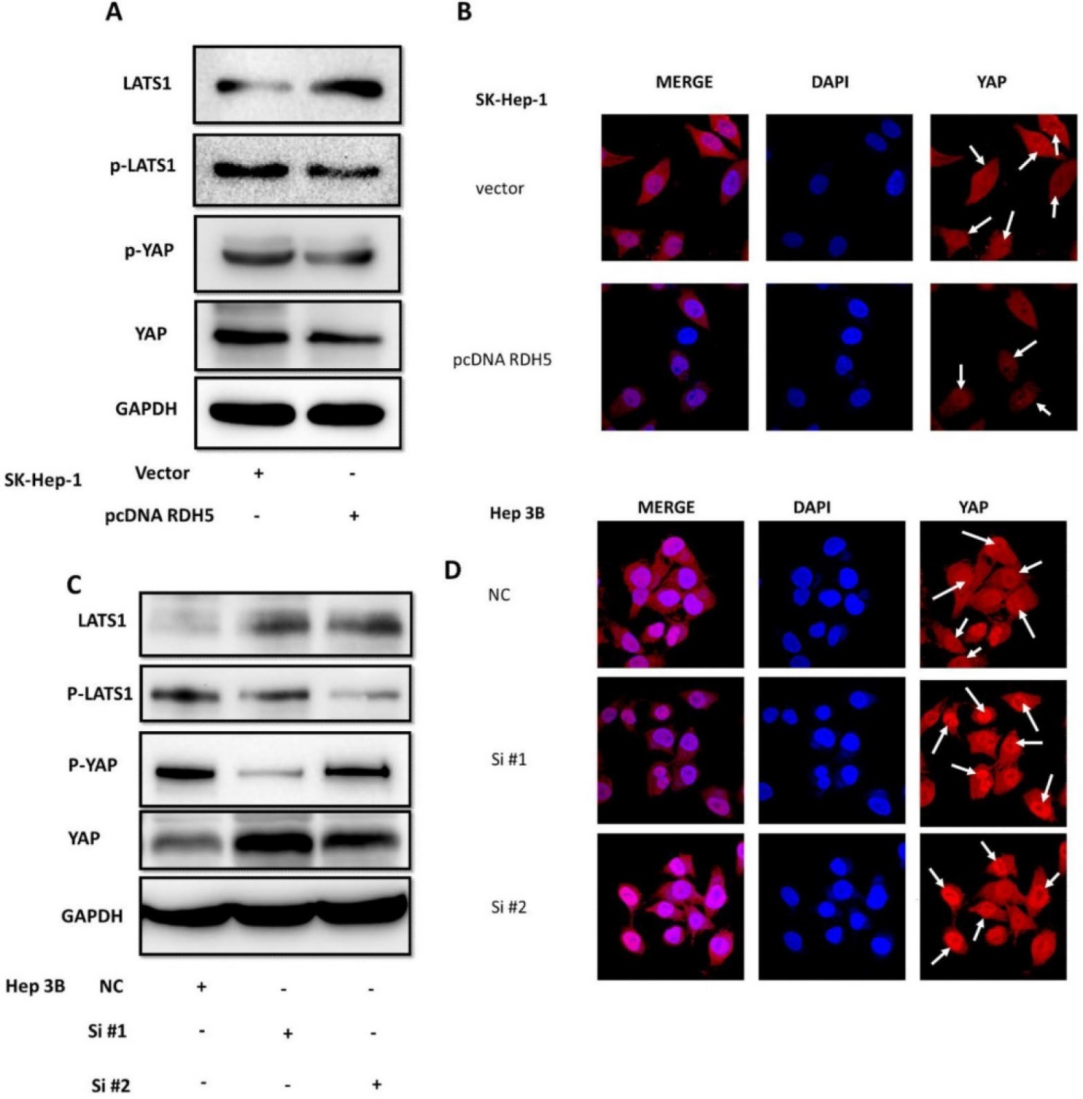
**RDH5 inhibits HCC cellular migration through suppressing Hippo/YAP pathway and changes the location of YAP.** Western blotting analysis of LATS1, p-LATS1, YAP, p-YAP expression levels after overexpression of RDH5 in SK-Hep-1 **(A)** and silencing of RDH5 in Hep 3B **(C).** Up-regulation of RDH5 suppress YAP protein into the nucleus in SK-Hep-1(the White arrow)** (B)**, Down-regulation of *RHD5* in hepatocellular carcinoma could promoting critical protein YAP step into the nucleus in Hep 3B (the White arrow)** (D)**.

Yes-associated protein (YAP) is an important mediator of cell differentiation, proliferation, and angiogenesis. Upon examination of the role of YAP in the RDH5-mediated inhibition of HCC motility, we found that overexpression of RDH5 could increase phospho-LATS1 levels and result in degradation of downstream YAP compared with the vector control (Fig. 5A). After silencing RDH5 with siRNA in Hep 3B cells, phospho-LATS1 levels were suppressed, and the total YAP level was increased (Fig. 5C). Protein localization and expression were visualized by immunofluorescence, which showed that downregulating *RDH5* in Hepatocellular carcinoma could activate the Hippo/YAP signaling pathway and promote the nuclear translocation of the crucial protein YAP in Hep 3B cells (Fig. 5D); the upregulation of RDH5 by plasmid transfection into SK-Hep-1 cells suppressed the protein expression of YAP, which remained in the cytoplasm rather than translocating into the nucleus (Fig. 5B).

### The low-level RDH5 expression is an independent, unfavorable prognostic role in hepatocellular carcinoma

To investigate whether *RDH5* is an independent factor in hepatocellular carcinoma in the entire study population, univariate and multivariate Cox analyses were conducted using SPSS version 21; Table 2 presents the results. The univariate analysis showed that seven factors, including new neoplasms, M stage, N stage, T stage (T3 and T4), tumor invasion, clinical stage (III-IV), and lower *RDH5* expression were poor prognostic factors for OS in hepatocellular carcinoma patients. According to the multivariate analysis, M stage, clinical stage (III-IV) and downregulation of *RDH5* level were independent risk markers and associated with shorter overall survival.

## Discussion

The *RDH5* gene encodes 11-cis retinol dehydrogenase, which is the key enzyme in the oxidation of 11-cis retinol to 11-cis retina. Low expression or mutation of the gene might contribute to retinitis pigmentosa-caused eye diseases. Although few histological studies have reported the relationship between *RDH5* expression and cancer, some articles have reported that the dysregulation of *RDH5* was associated with colorectal cancer 17, gastric cancer 18, and thyroid carcinoma 30. In our analysis, the expression of RDH5 was also associated with the clinicopathological feature of metastasis of hepatocellular carcinoma. On the other hand, higher RDH5 gene expression indicated longer survival by Kaplan-Meier survival analysis. Univariate Cox analysis demonstrated that new neoplasm, M stage, N stage, T stage (T3 and T4), tumor invasion, clinical stage (III-IV), and lower RDH5 expression can play prognostic roles in overall survival in hepatocellular carcinoma. The multivariate Cox analysis confirmed that RDH5 expression, M stage, and clinical-stage play independent prognostic roles in overall survival in hepatocellular carcinoma. Another report demonstrated microarray, RNA-Sequence, and EST database analyses identified 32 genes involved in all-trans-retinoic acid generation in normal colon and tumor tissues, including genes in the RDH, ADH, AKR, and CYP families.

Interestingly, Kang et al. 18 reported that *RDH5* is mutated in early gastric cancer and may be involved in early carcinogenesis; however, we found that the mutation rate of this gene was low in HCC (data not shown). Another large-scale experimental approach was used to assess CpG islands and promoter regions, and the findings showed DNA methylation of *RDH5* and gene expression in thyroid carcinoma 30. Pyrosequencing and RT-PCR showed that the *RDH5* gene is hypomethylated and overexpressed in papillary thyroid carcinoma tissues compared with matched non-neoplastic adjacent tissues, indicating that DNA methylation may regulate the expression of the *RDH5* gene. Based on these results, we explored the DNA methylation of RDH5. After predicting the DNA methylation profile using an online biomedical tool, we found that the *RDH5* gene has a CpG island in the promoter domain within the untranslated region (UTR). Besides, we analyzed data in the TCGA database and found that DNA methylation may cause the dysregulation of *RDH5* gene expression, and data showed that cells were treated with 5-Aza confirmed the previous results. This study is the first time to indicate that DNA methylation affects *RDH5* expression in hepatocellular carcinoma.

Hepatocellular carcinoma is one of the most malignant tumors in the world, about 50% of affected individuals live in China, and HBV infection is the primary risk factor 29. Owing to poor prognosis and recurrence, HCC is the 3rd-leading cause of cancer-related mortality 3. Numerous biomarkers have been reported in recent years, but there are no effective therapeutic targets for clinical treatment. Therefore, it is an urgent need to identify reliable biomarkers and therapeutic targets to monitor HCC progression. When the *RDH5* gene was overexpressed, the HCC cell line SK-Hep-1 partially lost metastasis ability and showed decreased cell proliferation. Nevertheless, gene-silencing in the HCC cell line Hep 3B contributed significantly to cell metastasis and proliferation. Immunoblotting demonstrated that *RDH5* can decrease EMT-associated protein markers, such as E-cadherin and vimentin. We confirmed that low RDH5 expression promotes metastasis by affecting EMT and cell proliferation.

Many studies have demonstrated that the Hippo pathway plays a critical signaling role in liver size control and tumorigenesis. Nuclear YAP may contribute to hepatocellular carcinoma metastasis 31. When *RDH5* was overexpressed in SK-Hep-1 cells, total YAP protein levels decreased. Conversely, silencing *RDH5* in Hep 3B cells increased the total YAP protein levels. To confirm the YAP protein localization in the cell, an immunofluorescence assay was performed, and the results indicated that silencing *RDH5* promoted YAP activation in the nucleus and that overexpressing *RDH5* promoted YAP inactivation by phosphorylating. In our research, we first showed that low expression of *RDH5* in hepatocellular carcinoma could activate the Hippo/YAP signaling pathway and promote YAP translocation into the nucleus.

Collectively, our research showed that *RDH5* was dysregulated in hepatocellular carcinoma patients, and this dysregulation can indicate a worse prognosis. First, low RDH5 expression was associated with metastasis in hepatocellular carcinoma tissues. Univariate and multivariate Cox analyses identified three factors, M stage, clinical stage (III-IV), and downregulation of *RDH5* expression, as independent risk factors associated with shorter overall survival. In our research, we found that higher gene expression of *RDH5* inhibited metastasis and cell proliferation. In addition, we found that *RDH5* affected metastasis by regulating the Hippo/YAP signaling pathway.

In summary, our study is the first to elucidate the critical roles of RDH5 in regulating the HIPPO/YAP signaling pathway in hepatocellular carcinoma. Moreover, RDH5 severs as an independent prognostic factor for HCC. We believe that RDH5 is a potential molecular target for predicting, preventing, and treating HCC metastasis. Despite many innovations demonstrated in our research, there are some limitations as well. First, we did not obtain enough tissue samples to perform immunohistochemistry and did not acquire sufficient evidence from animal experiments. Second, we found out that RDH5 works in HIPPO/YAP signaling pathway, but we didn't discover the direct evidence to prove how to regulate in this pathway and the detailed molecular mechanism is still unclear, and further studies are needed.

## Supplementary Material

Supplementary tables.

## Figures and Tables

**Figure 6:**
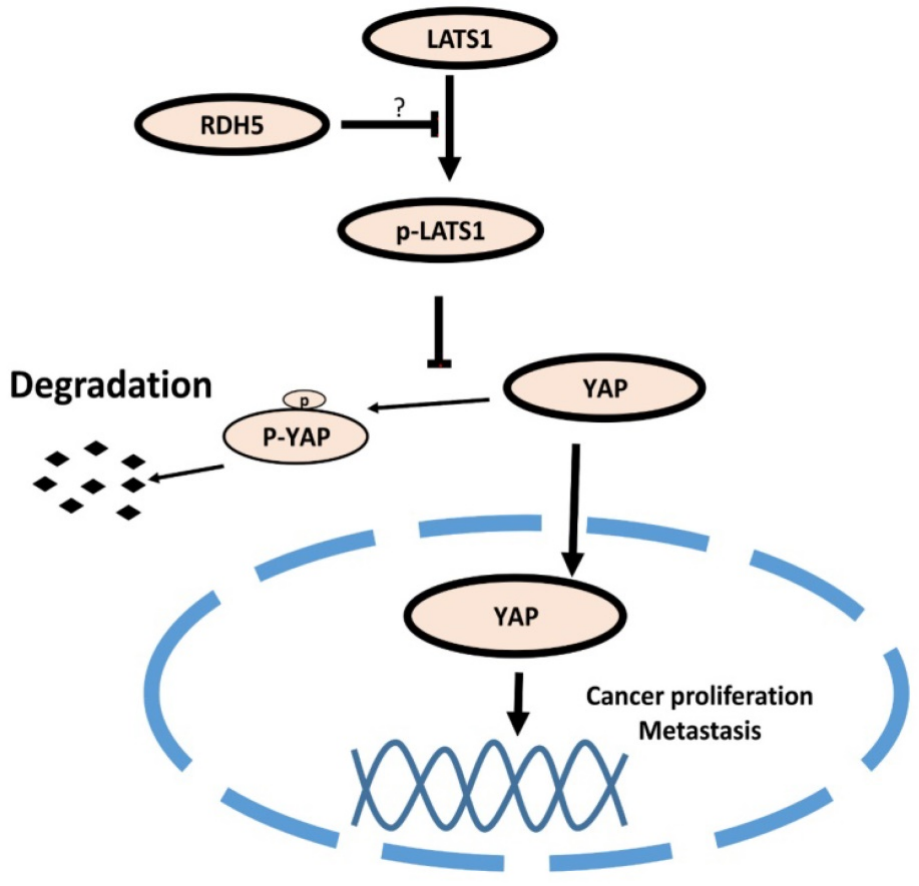
** Model of the RDH5 mechanism in HCC.** RDH5 promoting the (LATS1/2) can be phosphorylated and activated, phosphorylating and inactivating Yes-associated Protein, suppressing YAP into the nucleus.

**Table 1:** Correlation of RDH5 expression with clinicopathological features in HCC

Characteristics	Cases	RDH5 expression		*p* value
		High expression	Low expression	
**Gender**				0.481
Female	93	54	39	
Male	204	139	65	
**Age, years**				0.230
≤60	138	90	48	
>60	168	103	65	
**Child Pugh Classification**				0.324
A	182	115	67	
B	16	9	7	
C	10	9	1	
**AFP**				0.812
Negative	176	112	64	
Positive	130	81	49	
**Fibrosis**				0.794
Non-Fibrosis	51	29	22	
portal-Fibrosis	27	16	11	
Fibrosis-speta	21	16	5	
Nodular-Fibrosis	6	4	2	
Cirrhosis	57	38	19	
**Grade**				0.998
G1	39	24	15	
G2	151	95	56	
G3	100	63	37	
G4	11	7	4	
**M Stage**				0.023*
M0	226	151	75	
M1	70	42	38	
**N Stage**				0.139
N0	214	140	74	
N1-2	87	49	38	
**T Stage**				0.764
I	160	97	63	
II	71	45	26	
III	63	43	20	
IV	11	7	4	
**Clinical Stage**				0.555
I-II	215	136	79	
III-IV	71	47	24	
**Vascular history**				0.639
None	176	107	69	
Micro	73	49	24	
Macro	11	7	4	
**Year of diagnosis**				0.694
Before 2010	135	83	52	
After 2010	161	110	61	

The data are reported as number. P-value were obtained from the chi-square test.

**Table 2:** Univariate and multivariate COX analysis for RDH5 and survival of HCCs

Parameters	Univariate analysis		Multivariate analysis	
	HR (95% CI)	*p*	HR (95% CI)	*p*
**Gender**				
Male	Reference			
Female	0.78 (0.50-1.20)	0.260		
**Age, years**				
≤60	Reference			
>60	1.74 (1.10-2.74)	0.260		
**Child Pugh Classification**				
A	Reference			
B	1.18 (0.42-3.29)	0.751		
C	2.25 (0.31-16.47)	0.425		
**Grade**				
G1	Reference			
G2	0.86 (0.45-1.65)	0.648		
G3	1.09 (0.56-2,13)	0.806		
G4	1.49 (0.48-4.68)	0.490		
**AFP**				
Negative	Reference			
Positive	1.35 (0.88-2.07)	0.167		
**New Neoplasm**				
No	Reference			
Yes	0.58 (0.36-0.93)	0.025*		
**M Stage**				
M0	Reference			
M1	2.17 (1.39-3,37)	0.001*	2.09 (1.13-3.89)	0.019*
**Embedded**				
No	Reference			
Yes	0.49 (0.31-74)	0.001*		
**N Stage**				
N0	Reference			
N1	1.88 (1.21-2.94)	0.005*		
**T Stage**				
T1	Reference			
T2	1.45 (0.84-2.51)	0.186		
T3	2.32 (1.38-3.90)	0.002*		
T4	6.32 (2.77-14.45)	0.000*		
**Clinical Stage**				
I	Reference			
II	1.50 (0.84-2.69)	0.169		
III	2.30 (1.34-3.94)	0.002*	3.03 (1.62-5.67)	0.001*
IV	4.57 (1.39-15.09)	0.013*	4.91 (1.08-22.31)	0.040*
**Vascular invasion**				
No	Reference			
Yes	1.33 (0.90-1.96)	0.159		
**RDH5**				
High expression	Reference			
Low expression	1.78 (1.15-2.72)	0.007*	1.94 (1.15-3.28)	0.013*

NOTE: The entire clinicopathological variables lists in the table were included in the univariate and multivariate analysis. Abbreviation: 95%CI, 95%confidence interval.
